# Supplementary material for: Dynalign II: common secondary structure prediction for RNA homologs with domain insertions
Source: Nucleic Acids Res. 2014 Nov 21;42(22):13939–48. doi: 10.1093/nar/gku1172 (PMC4267632; doi:10.1093/nar/gku1172)
Supplement: SUPPLEMENTARY DATA [file supp_gku1172_nar-02021-z-2014-File012.zip › manual/RNA_class/html/_r_n_a_8cpp.html]

RNAstructure Classes: F:/RNAstructure-co-from-repository/RNAstructure/RNA\_class/RNA.cpp File Reference

- Main Page
- Classes
- Files

- File List
- File Members

# F:/RNAstructure-co-from-repository/RNAstructure/RNA\_class/RNA.cpp File Reference

`#include "RNA.h"`  
`#include "../src/structure.h"`  
`#include "../src/arrayclass.h"`  
`#include "../src/dotarray.h"`  
`#include "../src/forceclass.h"`  
`#include "../src/rna_library.h"`  
`#include "../src/stackclass.h"`  
`#include "../src/stackstruct.h"`  
`#include "../src/algorithm.h"`  
`#include "../src/outputconstraints.h"`  
`#include "../src/pfunction.h"`  
`#include "../src/boltzmann.h"`  
`#include "../src/alltrace.h"`  
`#include "../src/stochastic.h"`  
`#include "../src/MaxExpect.h"`  
`#include "../src/probknot.h"`  
`#include <iostream>`  

|  |  |
| --- | --- |
|  |  |
| Functions | |
| void | errmsg (int err, int erri) |
| Variables | |
| const float | epsilon = 1e-6 |

---

## Function Documentation

|  |  |  |  |  |
| --- | --- | --- | --- | --- |
| void errmsg | ( | int | *err*, |  |
|  |  | int | *erri* |  |
|  | ) |  |  |  |

---

## Variable Documentation

const float epsilon = 1e-6

---

Generated on Tue Jul 30 10:52:29 2013 for RNAstructure Classes by 
 1.5.7.1
